# Melioidosis molecular diagnostics: An update

**DOI:** 10.1080/21505594.2025.2505698

**Published:** 2025-06-14

**Authors:** Prachi Gangil, Manash K. Paul, Prathyoosha B, Debadrita Mondal, Sneha Kumari, P. R. Prasad, Vandana K. E., Bharti Bisht, Chiranjay Mukhopadhyay

**Affiliations:** a Department of Microbiology, Kasturba Medical College, Manipal Academy of Higher Education, Manipal, India; b Department of Radiation Biology and Toxicology, Manipal School of Life Sciences, Manipal Academy of Higher Education, Manipal, India; c Division of Pulmonary and Critical Care Medicine, David Geffen School of Medicine, University of California Los Angeles (UCLA), Los Angeles, CA, USA

**Keywords:** Melioidosis, diagnostics, early detection, point-of-care-test, liquid biopsy, artificial intelligence

## Abstract

Melioidosis, a fatal tropical disease, presents a wide array of clinical manifestations, including abscesses, pneumonia, septic shock, bacteraemia, osteomyelitis, septic arthritis, and skin infection. The Centers for Disease Control and Prevention (CDC) has classified *Burkholderia pseudomallei* (*B. pseudomallei*), a gram-negative bacterium found in soil, as a Tier 1 select agent. Referred to as the “great mimicker,” this organism can infect several organs imitating the symptoms of different illnesses. According to worldwide data, there are around 165,000 cases and 89,000 deaths annually. Current diagnostic procedures rely primarily on culturing *B. pseudomallei*, are slow and have low sensitivity, resulting in delayed treatment and higher fatality rates. This review examines the substantial difficulties related to diagnosing melioidosis in response to the urgent need for precise and prompt diagnosis. We have summarized the results of diagnostic kits that are currently sold in the market and assessed the market for melioidosis diagnostic kits.

## Introduction

Melioidosis, also known as Whitmore’s disease, is a neglected tropical disease caused by motile, gram-negative bacteria; *Burkholderia pseudomallei* (*B. pseudomallei*), which is found in contaminated soil and water mainly in tropical and subtropical regions such as northern Australia and southeast Asia. It is also endemic along the coastal region of south India [1]. The common reservoirs for *B. pseudomallei* are rice paddy fields, stagnant water, moist tropical soil, and roots of plants. This bacterium is reported to persist for extended periods under low-nutrient conditions. The best growth is found in soil with a water content of 15%, and most infections occur during the rainy season when bacteria leached from the soil and came to the surface [2]. Up to 1,65,000 cases are reported worldwide for melioidosis, out of which 89,000 cases are fatal [3]. This number is far from the actual value because many cases remain unreported due to several diagnostic challenges [4]. The disease affects humans and animals, including non-human primates and wild and domestic animals. However, direct transmission of infection between humans and animals is not common [5]. Infection is reported to occur by direct contact of bacteria to the host via inhalation, ingestion, and percutaneous inoculation. Human-to-human transmission of *B. pseudomallei* is quite rare. Sexual transmission has been proposed but not conclusively confirmed as a route of infection [6].

*B. pseudomallei* has a large genome size of 7.2 Mb that encodes for several virulence factors like Type III Secretion System (T3SS), Type VI Secretion System (T6SS), autotransporter proteins, polysaccharides, lipopolysaccharide (LPS), flagella, etc. These virulence factors assist the bacteria in adhering to and invading the host cells, multiplying within hosts, infecting neighbouring cells, and overcoming several antimicrobial host defence systems. Following the invasion, *B. pseudomallei* escapes the host’s intracellular bacteria-killing phagosome-mediated mechanism using proteins like BopA, TT3SS, bsaZ, etc [7]. Bacterial virulence factors manipulate the host cells and undergo intracellular replication inside the host. Type IV Secretion System (T4SS) plays an important role in the fusion of host cells to form the multinucleated giant cells (MNGCs) by polymerization of host cell actin, which facilitates cell-to-cell dissemination [8]. *B. pseudomallei* is known to replicate in phagocytic and non-phagocytic cells [9], producing different types of exotoxins and endotoxins that cause infection [10]. Toll-like receptors (TLRs) recognize pathogen-associated molecular patterns (PAMPs) like LPS, flagella, etc., and mediate Nuclear factor kappa B (NF-κB) mediated activation of immune response by releasing pro-inflammatory cytokines like Interleukin-1 beta (IL-1β), and Interleukin-18 (IL-18). Intracellular inflammasome receptors also recognize bacterial virulence factors and damage-associated molecular patterns (DAMPs) that trigger caspase 1-mediated pyroptosis and further release IL-1β and IL-18, which ensures the formation of IFN-γ. Neutrophils, dendritic cells, B cells, and T cells are reported to be recruited at the site of infection, which leads to the activation of complement and coagulation cascades [11].

Treatment regimens for melioidosis are often thought of as consisting of two phases, according to the current convention: An initial intensive/acute phase followed by an eradication phase. The acute phase, which aims to save patients from fatality due to disseminated infection, and the latter one is the eradication phase, which aims to kill the residual bacteria inside the body or prevent relapse. Ceftazidime (CAZ) and Carbapenems are two common antibiotics administered to the patient during the acute phase. Trimethoprim-sulfamethoxazole (SXT) or Amoxicillin-clavulanic acid (AMC) are administered later, i.e. in the eradication phase. *B. pseudomallei* expresses chromosomally encoded resistance determinants, increasing antibiotic resistance likelihood. Therefore, it is necessary to conduct antibiotic susceptibility monitoring to identify the most efficient antibiotics for treating melioidosis [4].

In this review, we provided comprehensive information on several approaches for diagnosing melioidosis, including radiological imaging, fine-needle aspiration, liquid biopsy, and metabolite profiling, which are used to identify particular biomarkers for diagnosis. Furthermore, we have summarized the outcomes of diagnostic kits now available on the market. The methodology for finding the available data involved a systematic search of the PubMed database to gather relevant literature. The primary keywords included “melioidosis,” “Burkholderia pseudomallei,” “diagnostic methods,” “early detection,” “symptoms,” “detection challenges,” molecular diagnostics“, “PCR,” “ultrasound,” “X-ray,” “antibiotic resistance,” “point-of-care testing,” “liquid biopsy,” “extracellular vesicles,” “cell-free DNA,” “serological assays,” “clinical manifestations,” “market,” and “diagnostic kits.” The search was restricted to articles published in English, which were screened for relevance through title and abstract evaluation, followed by a full-text review of selected studies to extract pertinent data. We also did literature searches for patents filled (https://www.uspto.gov/) in this area. We have assessed the current market conditions for melioidosis diagnostic kits, considering technological advancements and challenges in meeting the growing need for rapid and accurate diagnostic solutions.

## Melioidosis is a deadly disease

*B. pseudomallei* is classified as Tier 1 Select Agents by the Centers for Disease Control and Prevention (CDC), recommending handling it within a class II biosafety cabinet in a Biosafety Level 3 (BSL3) facility [8]. The fatality rate lies between 10% and 50 %, but due to delays in timely diagnosis of the disease, the fatality rate can go as high as 90% in some endemic areas [5,9]. Misdiagnosis, lack of awareness, and unavailability of proper healthcare facilities play a significant role in increasing the fatality rate of melioidosis patients. Due to these diagnostic challenges, countries like Malaysia have been reporting a 65% mortality rate for several years [4]. Diabetes mellitus is one of the most remarkable predisposing factors for melioidosis, with approximately 45.68% (95% CI: 44.8–46.57, *p* < 0.001) of cases occurring in diabetic patients. A systematic review and meta-analysis revealed that individuals with diabetes are three times more likely to develop melioidosis compared to non-diabetic individuals (RR 3.40, 95% CI: 2.92–3.87, *p* < 0.001) [12]. Other contributing factors include age, exposure to contaminated soil and water, and pre-existing health conditions.

For instance, in northeast Thailand, the incidence rate of melioidosis per 100,000 for non-diabetics is 6.8, 145.7 for diabetics, and 84.4 in patients with undiagnosed diabetics, respectively [8]. People with pneumonia, multiple-organ involvement, or septicaemia have a greater mortality rate if compared with individuals with single organ involvement without pneumonia [13]. The global mortality rate of melioidosis is said to be higher compared to other infectious diseases like leptospirosis and dengue, but it still receives less attention and concerns [4].

## Melioidosis clinical signs and symptoms

Melioidosis is dubbed the “great mimicker” as it mimics the clinical symptoms of other diseases, hence making the diagnosis of the disease a significant challenge. The symptoms are reported to occur after 1–21 days post-exposure, with the clinical presentation varying according to the site of infection [5]. If symptoms persist for more than 2 months, then the infection is considered chronic, though acute symptoms are more commonly observed in patients [14]. *B. pseudomallei* causes a broad spectrum of symptoms that range from asymptomatic to local abscesses and lower respiratory tract infections [15]. The most common symptoms are fever, headache, respiratory distress, abdominal discomfort, joint pain/disorientation, etc., and other rare symptoms such as acute suppurative parotitis and lacrimal gland infection are also reported [4]. The incubation period of the disease is 1 day to 3 weeks. However, latent melioidosis can remain asymptomatic for decades. The disease can occur acutely or chronically with highly variable symptoms ranging from localized infections to abscesses of the liver, spleen, prostate, or parotid glands to sepsis [10]. Melioidosis can potentially impact all organs of the body but is found to be most frequently associated with pulmonary infections, mimicking pneumonia-like symptoms such as cough, high fever, chest pain, etc [13]. In endemic areas, ocular melioidosis can be an important disease manifestation. In musculoskeletal melioidosis, sternoclavicular joints can be involved in disseminated form [16]. The knee joint is also reported to be commonly affected, followed by the ankle, hip, and shoulder. In Australia, encephalomyelitis is the most frequent manifestation of neuro-melioidosis [17]. Osteomyelitis and septic arthritis are also reported, which might be due to penetrating injuries or blood dissemination [14].

## Challenges of Melioidosis

### Detection

**Figure 1.**
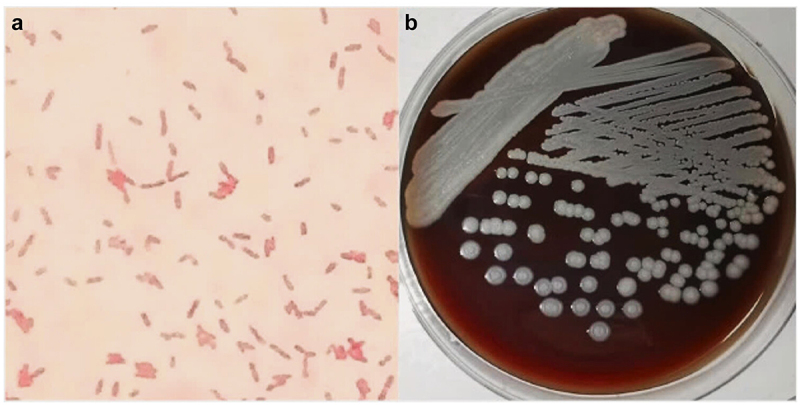
The morphological characteristics of *B. pseudomallei* and its colony as cultured on blood agar plates. *B. pseudomallei* is a Gram-negative, rod-shaped bacterium: (a) The results of Gram staining for *B. pseudomallei* reveal pink-stained cells under the microscope, one of its distinctive features is the “safety pin” structure, which is due to bipolar staining; (b) The colonies are usually cream to white in colour, but over time they may develop a characteristic wrinkled appearance when incubated for longer duration. Used with permission (doi .Org/10 .1016/j.Heliyon.2024.e30299).

Cases of melioidosis are highly underreported worldwide. This is because clinical diagnosis of melioidosis can be challenging due to its non-specific symptoms, leading to misdiagnosis and delayed treatment. Moreover, due to its resemblance to *Pseudomonas spp.*, it might be neglected as a common laboratory contaminant in microbiology labs. The current gold standard for melioidosis detection is based on culturing *B. pseudomallei* from patient samples. Most commonly, the morphological characteristics of *B. pseudomallei* and its colony as cultured on blood agar plates is investigated. One of its distinctive features is the “safety pin” structure, which is due to bipolar gram staining (Figure 1). However, this process is very time-consuming and can take up to 7 days to yield results. In addition to this delay, the culture offers poor sensitivity [18]. Despite these shortcomings, culturing *B. pseudomallei* on a selective agar continues to be used as the gold standard due to the lack of better alternatives. Ashdown’s medium is a selective medium used in endemic countries for selective isolation of *B. pseudomallei*. Before Ashdown’s medium, initial isolation relied on standard blood, MacConkey, and chocolate agars, which often resulted in misidentification of *B. pseudomallei* as contaminants or *Pseudomonas* species due to their similar morphology. Ashdown’s medium, introduced in 1979, includes crystal violet and gentamicin to selectively promote the growth of *B. pseudomallei*, which produces distinctive purple, dry, and wrinkled colonies after 48–96 hours of incubation. The *B. pseudomallei* selective agar (BPSA), utilizing maltose and Nile blue while excluding crystal violet, accelerates colony differentiation with faster growth compared to Ashdown’s agar. Francis medium enhances differentiation between *B. pseudomallei* and *B. cepacia*, achieving 78.4% sensitivity and 92.2% specificity *in vitro*. A modified Ashdown’s agar consisting of norfloxacin, ampicillin, and polymyxin B was reported to improve specificity in detecting *B. pseudomallei* from gastrointestinal samples in a mouse model [3].

Differences in clinical symptoms among melioidosis patients might be observed across different geographical regions. Therefore, it is crucial for clinicians to be aware of the common presentations in their specific location. For example, in Australia, melioidosis is accompanied with a high incidence of genitourinary infections along with prostatic abscesses. Encephalomyelitis with flaccid paralysis is also observed among the patients from this region. On the contrary, if we compare the clinical manifestations of the patients in Thailand, liver and splenic abscesses along with suppurative parotitis are more commonly observed. Hence, making it challenging for the clinicians to develop a standardized symptom guide for melioidosis patients.

**Figure 2.**
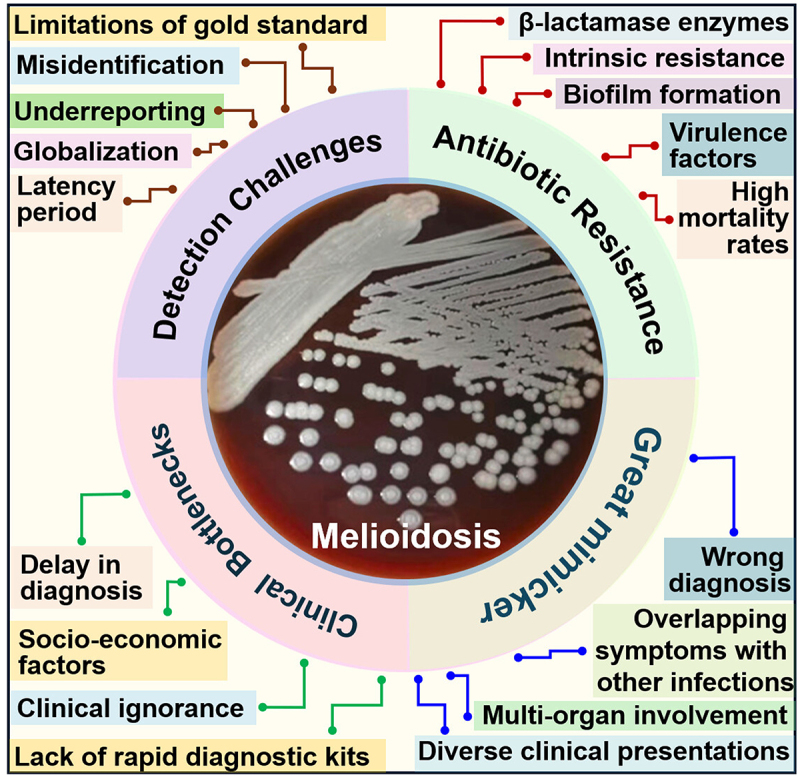
Challenges of Melioidosis. Melioidosis caused by *B. pseudomallei* is highly underreported worldwide. The reasons for its underdiagnosis are multifactorial. Their resistance to common antibiotics and non-specific symptoms that mimic the symptoms of other diseases lead to misdiagnosis and delayed treatment. Moreover, the gold standard of disease involving bacterial culture is slow and requires specialized conditions, leading to high mortality rates. The limited availability of advanced molecular diagnostics in endemic regions further complicates timely and accurate detection.

Although this disease can infect individuals of any age group, the incidence rate peaks among individuals belonging to 40–60 years of age. Furthermore, the disease is more commonly observed in males due to their involvement in occupations that require them to be more frequently in contact with soil [19]. Another major issue related to the detection of melioidosis is its long latency period. Some individuals may carry the bacteria and still be asymptomatic, making it difficult to detect the infection early. The average incubation period among melioidosis patients is usually mean 9 days. However, the most prolonged reported incubation period is 62 years, hence the nickname “Vietnamese time bomb” [20]. Although melioidosis is endemic to southeast Asia and northern Australia, global warming and mass international travel to these countries have increased disease incidence in non-endemic regions. Lack of awareness about the clinical presentations and risk factors among the healthcare providers of this region hinders the timely detection of the disease [21]. The challenges of melioidosis detection is shown in Figure 2.

### Antibiotic resistance

**Figure 3.**
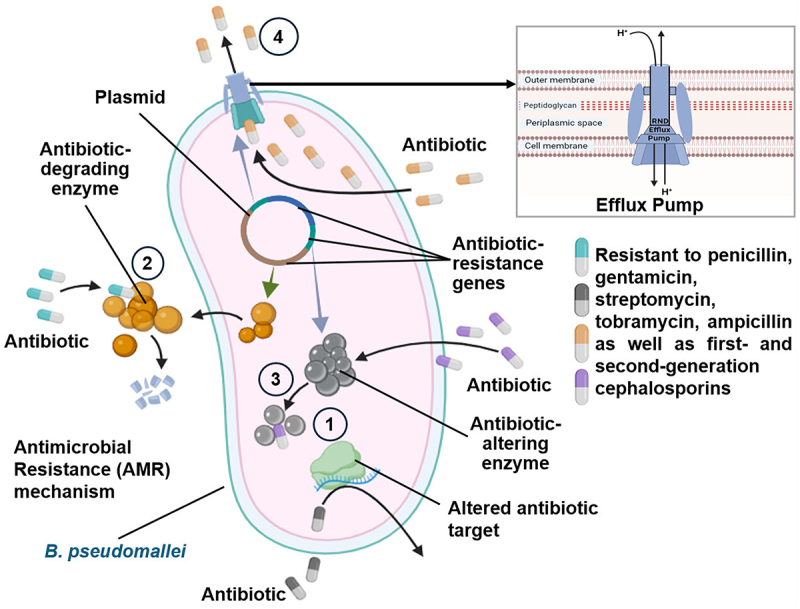
Antibiotic resistance mechanism in *B. pseudomallei*. *B. pseudomallei* exhibits intrinsic resistance to a range of antibiotics such as penicillin, gentamicin, streptomycin, tobramycin, ampicillin, and first- and second-generation cephalosporins. This resistance is mediated through several mechanisms: 1) Alteration of an antibiotic target site, thereby reducing drug binding efficacy; 2) Degradation of antibiotics by β-lactamase enzymes, hydrolysing the antibiotics containing β-lactam rings; 3) Inactivation of antibiotics by the antibiotic altering enzymes; 4) Efflux of antibiotics reduce the intracellular antibiotic concentration by RND (Resistance-nodulation-division) efflux pumps.

*B. pseudomallei* is known to exhibit intrinsic resistance to various antibiotics such as penicillin, gentamicin, streptomycin, tobramycin, and ampicillin, as well as first- and second-generation cephalosporins. Intrinsic resistance is associated with multiple factors, consisting of antimicrobial cell entry, expulsion, and enzymatic degradation. Among these, the most clinically relevant are efflux pumps of the resistance nodulation cell division (RND) family. The genome of *B. pseudomallei* codes for around ten RND efflux pumps, out of which seven are located on chromosome 1 and the remaining three on chromosome 2 [3]. Biofilm formation is another crucial mechanism of antibiotic resistance in *B. pseudomallei* [22]. It provides a protective environment for the bacteria, thereby reducing the penetration power of antibiotics and other antimicrobial agents by creating a physical barrier, shielding the bacteria from the host’s immune system. Therefore, compared to free-living bacteria, biofilm-associated bacteria are reported to be more antibiotic-resistant. The biofilm matrix formed by *B. pseudomallei* includes a crucial component for biofilm establishment: Extracellular DNA (eDNA). However, the mechanism by which eDNA confers antibiotic resistance to *B. pseudomallei* remains unclear [23]. The genome of *B. pseudomallei* also codes for several types of β-lactamase enzymes such as Ambler class A, B, and D β-lactamases. These enzymes are responsible for breaking down β-lactam antibiotics, making them ineffective. The most important gene among these is penA, located on chromosome 2. This gene produces a class A lipoprotein attached to the cell membrane and secreted by the twin-arginine transport system, helping the bacteria to resist and hydrolyse most β-lactam antibiotics [11]. The antibiotic resistance mechanism in *B. pseudomallei* is shown in Figure 3. The antibiotic resistance patterns in *B. pseudomallei* are not uniform but vary considerably across different parts of the world. This variability underscores the need for localized surveillance and tailored treatment strategies to effectively manage infections caused by this pathogen. Table 1 presents a brief summary of the antibiotic resistance profiles of *B. pseudomallei* reported across various countries worldwide.

**Table 1.** Global perspective of antibiotic resistance and susceptibility patterns in *B. pseudomallei*.

Country	Meropenem	Ceftazidime	Imipenem	Amoxicillin/Clavulanate	Trimethoprim/ Sulfamethoxazole (TMP-SMX)	Doxycycline/Tetracycline	Ref.
Malaysia	100% susceptible to antibiotic	0.6% is resistant	0.6% is resistant	0.6% is resistant	10% is resistant	0.6% is resistant	[24]
Bangladesh	100% susceptible	100% susceptible	100% susceptible	100% susceptible	*B. pseudomallei* is sensitive	100% susceptible	[3,24]
Australia	100% susceptible to antibiotic	4% *is* resistant	4% *is* resistant	Resistant	4% *is* resistant	4% is resistant	[24,25]
Thailand	100% susceptible to antibiotic	0.6%*i* is resistant	No resistance detected	0.6% is resistant	0.6% is resistant	0.6% is resistant	[3,24,26]
Southern Taiwan	100% susceptible to antibiotic	100% susceptible to antibiotic	100% susceptible to antibiotic	93% susceptibility to antibiotic	100% susceptible to antibiotic	100% susceptible to antibiotic	[27]
China (Hainan)	100% susceptible to antibiotic	12.8% increase in resistance	–	–	–	–	[24,28]
Loas	100% susceptible to antibiotic	–	–	–	99.2% susceptibility to antibiotic	–	[3]
Cambodia	100% susceptible to antibiotic	–	–	–	100% susceptible to antibiotic	–	[3]
Brazil	100% susceptible to antibiotic	–	–	–	100% susceptible to antibiotic	–	[3]
Singapore	–	0.5% is resistant	–	–	–	–	[3]
India	susceptible	susceptible	–	–	susceptible	resistance	[29]

The bacterium also possesses several virulence factors, further complicating treatment options and leading to severe disease outcomes. Despite this, antibiotics are the backbone of melioidosis treatment to date due to the lack of research initiatives in exploring alternative treatment options. The mortality rate can be reduced to 10% with timely antimicrobial treatment. However, in poorly resourced areas, the mortality rate can still be as high as 50% [30]. Although uncommon, the bacteria can develop acquired resistance against clinically significant drugs at a later stage, leading to failure of antibiotic treatments or substantial mortality rate in the patients [31]. The challenges associated with antibiotic resistance and melioidosis is shown in Figure 2.

### Early diagnosis

The major problem associated with early diagnosis of melioidosis infection is clinical ignorance by healthcare providers and misidentification of *B. pseudomallei* as some other bacteria or a common laboratory contaminant. Another major reason for melioidosis being neglected is that, unlike COVID-19, it is unlikely to spread from human to human. Hence, proper control measures should be taken to reduce the incidence and mortality rate of this infectious disease [32]. Early diagnosis of melioidosis is also necessary because, if delayed, bacteraemia can occur in 40–60% of the patients. Likewise, 20% of the patients can develop septic shock, which is considered to be the most severe form of the disease [33]. Treatment delays can arise from various factors, including challenges in clinical recognition due to non-specific symptoms and the extended duration required for laboratory diagnosis. This can lead to poor patient outcomes, where the mortality rate can increase to as high as 40% in some regions [11]. For this reason, there is an urgent need to increase awareness about melioidosis among the community, especially those living in endemic regions, and realize its actual burden within our community. Thus, better diagnostic tests and early confirmation of the diagnosis are critical. This will aid in improving the survival rate of patients with better therapeutic efficacy. A recent case study of a diabetic Sri Lankan male presenting symptoms with fever and multiple abscesses in the liver, spleen, and psoas muscle was initially misdiagnosed with tuberculosis, delaying the identification of melioidosis [34]. This case highlights the critical need to include melioidosis as a differential diagnosis in immunocompromised patients with multi-organ abscesses. Early detection of such abscesses enables prompt antibiotic treatment, improving patient outcomes.

Melioidosis predominantly affects rural populations with poor socioeconomic backgrounds in low- and middle-income countries where the patients approach the healthcare facilities in the terminal phase of the disease. These regions often lack adequate laboratory facilities for early detection and diagnosis of the disease, contributing to delayed diagnoses. Consequently, many cases progress to severe stages, resulting in higher mortality rates. The scarcity of medical resources, combined with limited access to healthcare, exacerbates the impact of melioidosis, leading to a disproportionate number of deaths in low-income countries [32]. Furthermore, several risk factors associated with melioidosis infection should be taken into consideration when making the diagnosis. These include diabetes, chronic liver and renal failure, alcoholism, malignancy, old age, and agricultural activities [12]. The challenges of melioidosis early diagnosis is shown in Figure 2.

### Great mimicker

Melioidosis is often referred to as the “great mimicker” due to its ability to present itself with a wide range of clinical manifestations, complicating the correct diagnosis of the disease [35]. Melioidosis is associated with multiorgan involvement, with lungs being the most common site of infection [13]. Among the abdominal visceral organs, the spleen and liver are most commonly involved and are characterized by abscesses with a typical “honeycomb pattern” [35]. Musculoskeletal manifestation includes soft tissue abscesses, septic arthritis, and osteomyelitis [36]. In addition, the infection can also spread to other parts of the body, such as the prostate, brain, and kidney, demonstrating its diverse and complex nature [9]. The disease can follow the clinical course of asymptomatic, acute, chronic, subacute, or a very long latent phase, resembling other common infections [3]. *B. pseudomallei* is also known to possess broad-spectrum antibiotic resistance and multiple virulence factors, enabling it to produce various non-specific signs and symptoms due to its high adaptability and hindering the correct diagnosis [35]. In more than half of the melioidosis patients, pneumonia is reported as the primary symptom. Patients may exhibit fever, cough, chest pain, and lung abscesses [37]. In countries like India, where tuberculosis is endemic, chronic melioidosis can be easily misdiagnosed as tuberculosis. They share symptoms like chronic cough, weight loss, pulmonary cavitations, and night sweats [38]. Hence, an early clinical suspicion aided by early diagnostic kits is necessary to make the correct diagnosis.

Melioidosis being a multi-systemic infection, it is crucial to combine imaging results with laboratory tests before excluding the possibility of melioidosis as it can lead to misdiagnosis [39]. Arunpriyandan V. *et al*. (2022) reported a case where an adult diabetes mellitus patient complained about acute knee joint pain and swelling. However, he was subsequently diagnosed with melioidosis with septic arthritis as the major clinical presentation [40]. In another case study, it was found that even though liver and splenic abscesses can be caused by several organisms such as *Mycobacterium tuberculosis, Candida albicans*, etc., to name a few, the presence of splenic and hepatic abscesses together, especially in patients with a history of travel to endemic countries or having underlying comorbidities is highly suggestive of melioidosis infection [35]. Long F. *et al*. (2022) reported a case study where even though medical imaging data suggests the diagnosis to be a multi-systemic malignancy, laboratory tests indicated the presence of melioidosis infection [39]. Huang Wy. *et al*. (2018) reported a case study involving the central nervous system (CNS) during melioidosis infection. In the early stages, CNS melioidosis can manifest symptoms similar to stroke or malignancy, leading to a false diagnosis [41]. Therefore, healthcare workers need to put more effort into accurately diagnosing the disease before a treatment plan is selected, suggesting the need for a Point-of-Care-Test (POCT). The clinical bottlenecks and treatment challenges of melioidosis as a great mimicker are shown in Figure 2.

## Requirement of point-of-care-test

A POCT is a rapid diagnostic method that provides immediate results without requiring extensive laboratory facilities. It is a sensitive and specific test that helps diagnose a patient’s disease and enables continuous monitoring and effective management of the patient’s condition. POCT is also incredibly beneficial for effective patient triage in emergencies, reducing complications related to delayed diagnosis and facilitating timely treatment to the patients. According to the World Health Organization (WHO), the criteria for an ideal POCT include easy accessibility, affordability, sensitivity, specificity, rapidity, robustness, equipment-freeness, and user-friendliness to end users [42]. Technological advancements have revolutionized POCT, allowing the possibility of miniaturization of electronics to improve instrumentation, enabling the development of more accurate POCT devices that are much smaller in size [43]. The urgency for a POCT for melioidosis is evident due to the limitations of the current diagnostic methods. POCTs can provide rapid and accurate results, allowing for immediate initiation of appropriate treatment. This is crucial in preventing the high mortality rate associated with melioidosis, especially in resource-limited settings where access to sophisticated laboratory facilities is limited [44]. Implementing POCT for melioidosis offers several advantages, including rapid diagnosis, early initiation of targeted therapy, and the potential to reduce the spread of the disease in endemic regions. Furthermore, it can streamline patient management and improve clinical outcomes, ultimately reducing the burden on healthcare systems.

**Figure 4.**
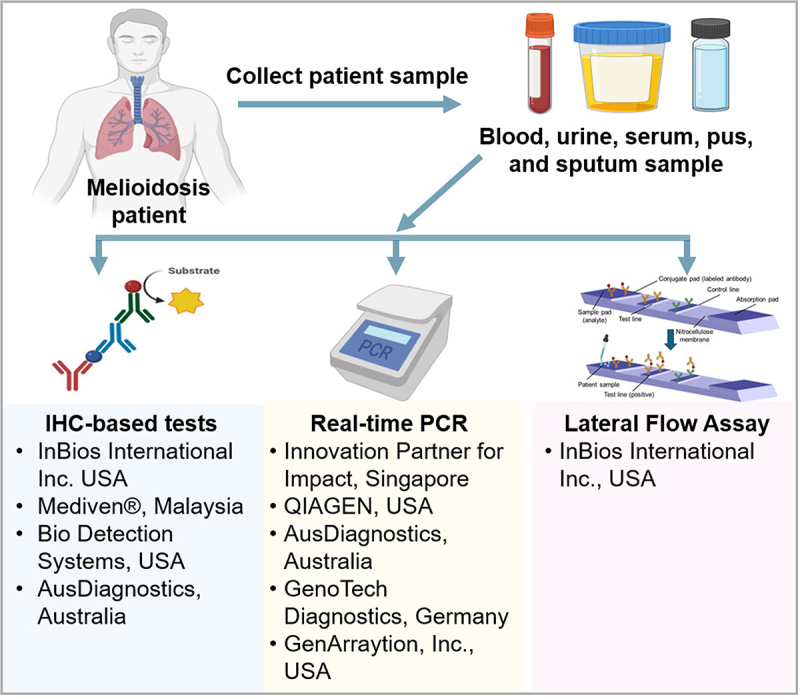
Development of diagnostic kit for melioidosis detection. The current diagnostic kits for melioidosis are primarily based on the following technologies: Immunohistochemistry, real-time PCR, and lateral flow assays. These assays are tailored to detect patient samples (blood, urine, sputum, etc.) containing specific biomarkers associated with the disease. Rigorous validation procedures are conducted to optimize sensitivity and specificity, culminating in producing prototype kits designed for precise and reliable detection of melioidosis. The major companies involved in developing and producing these diagnostic kits are also highlighted for each category.

To address the urgent need for a POCT, it is essential to understand that a delay in diagnosis and initiation of appropriate treatment significantly contributes to adverse outcomes for patients with melioidosis, leading to a high mortality rate. Furthermore, the challenges posed by the current diagnostic methods, including the need for specialized laboratory facilities and the time-consuming nature of culturing the bacterium, highlight the critical importance of a rapid and accessible POCT. The guidelines for development of a POCT kit for melioidosis detection using different techniques is shown in Figure 4. The necessity can be further emphasized by the potential for melioidosis to cause sepsis [45] and the lack of specific early clinical features, which often lead to misdiagnosis or delayed diagnosis. Additionally, in resource-limited settings, where the burden of melioidosis is exceptionally high, the availability of a POCT could significantly impact patient outcomes and public health by enabling timely and targeted interventions. Considering these pressing concerns, it is evident that developing and implementing a POCT for melioidosis is paramount in addressing the urgent need for rapid and accurate diagnostic solutions in endemic regions or even non-suspecting, non-endemic regions.

## Available melioidosis detection methods

**Figure 5.**
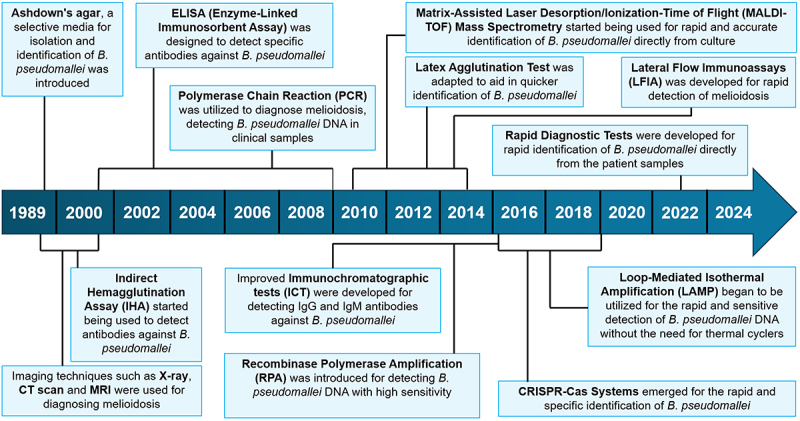
Milestones in melioidosis detection techniques. Significant progress has been made in the detection techniques for melioidosis. This timeline highlights the chronological account of important milestones in the evolution of diagnostic methods for melioidosis. Over the decades, diagnostic approaches have progressed from basic culture-based methods and serological assays to more precise biochemical tests and PCR-based molecular techniques. The development of rapid diagnostic tools and the adoption of next-generation sequencing have further enhanced diagnostic accuracy and strain typing, significantly improving the management and control of melioidosis.

**Figure 6.**
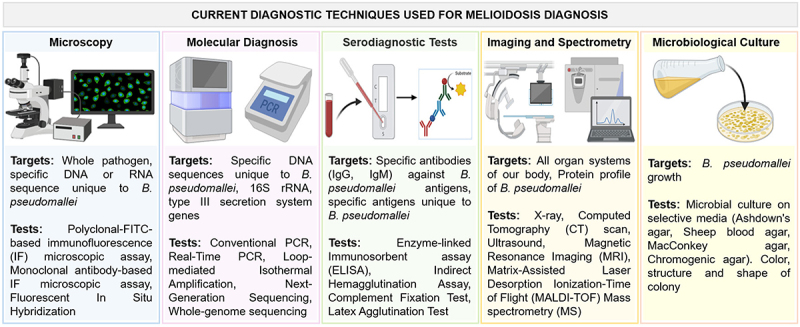
Current and future diagnostic techniques used for melioidosis diagnosis. This cartoon representation presents a comprehensive overview highlighting the progression from traditional methods to cutting-edge technologies, aiming to achieve earlier detection, improved accuracy, and better patient outcomes in melioidosis management. Existing techniques include bacterial culture, which remains the gold standard but is time-consuming, along with imaging studies and serological assays such as ELISA, immunohistochemistry, and molecular methods like real-time PCR, which offer higher sensitivity and specificity.

Despite the development of several melioidosis diagnostic tests, their overall sensitivities and specificities are often poor, and some lack standardization in clinical settings. The milestones in melioidosis detection techniques is presented in Figure 5. Several methods are currently used for the melioidosis detection and are summarized in Figure 6, and explained in the subsequent sub-sections.

### X-Ray/CT

Radiological imaging techniques can also be used for diagnosing melioidosis. Techniques such as X-rays, Computed Tomography (CT) scans, and Magnetic Resonance Imaging (MRI) scans can help identify melioidosis manifestations across different organs of the patient’s body [46]. Though melioidosis causes a multi-systemic infection; however, pulmonary melioidosis is the primary site of infection [47]. Therefore, with the help of these radiological imaging techniques, we can visualize the extent of infection in the body and verify if there is any involvement of multiple organ systems in the patients. Carrillo-Bayona JA. *et al*. (2021) reported the most common imaging findings of lungs seen in melioidosis patients. Chest X-ray results of *B. pseudomallei*-infected lungs showed consolidation in 86% of cases, nodules in 26%, and cavitation in 20% of the melioidosis patients. CT scans revealed consolidation and nodules in 90% of the patients, with areas of consolidation predominantly located in the basal and central zones in 60% of the cases [48]. Awareness of the potential radiological manifestations can guide clinicians in achieving early diagnosis and initiating appropriate treatment. There are no specific imaging results that could specifically differentiate *B. pseudomallei* infection from other lung infections. Generally, there are no known characteristic features in imaging that can specifically differentiate melioidosis from other infections. However, the “honeycomb” appearance has been described to be the characteristic feature of large liver abscesses in melioidosis patients. Another distinctive feature of melioidosis infection is the simultaneous involvement of multiple organs, as revealed by radiological imaging [49].

Bedside ultrasound is a crucial diagnostic tool for melioidosis in resource-limited settings, offering rapid and non-invasive identification of abscesses. In a study conducted in Laos, 61% of melioidosis patients had abscesses detectable via ultrasound with a high positive predictive value of 93% (88–96%) for Melioidosis [50]. While the absence of abscesses cannot exclude melioidosis suspicion, their presence should strongly prompt clinicians to consider a potential disease diagnosis.

### Fine needle aspiration

Fine Needle Aspiration (FNA) biopsy techniques have become valuable for detecting infectious diseases such as melioidosis. Mohan A. et al. (2021) conducted a retrospective descriptive study in Malaysia, where they described the usage of FNA with inoculation in a blood culture bottle as a valuable technique for detecting infection among children with melioidosis of head and neck. This technique of using blood culture bottles for culturing bacterial samples from non-blood specimens has been reported to improve the yield of *B. pseudomallei* isolated compared to the yield obtained from directly plating it on selective agar [51]. Although bacterial isolation by culture is the gold standard for diagnosing melioidosis, obtaining proper tissue samples from the infection site is also crucial for its early diagnosis (Figure 1). When the melioidosis manifestations involve deeper tissue, such as intra-abdominal organs and mediastinum, it might get challenging for clinicians to obtain tissue samples for laboratory testing from these remote regions. Subramanian S. et al. (2020) described a useful technique for early diagnosis of mediastinal melioidosis to solve this issue. They reported using endobronchial ultrasound (EBUS) guided FNA as a useful tool for locating and sampling mediastinal lesions [52].

### Liquid biopsy

Liquid biopsy involves the analysis of the components present in body fluids like blood, saliva, urine, and cerebrospinal fluid [53]. These components could be circulating cells, cell-free DNA, microRNA, exosomes, or other genetic material and molecules associated with the disease pathogenesis [54]. It can be applied to diagnose melioidosis by identifying circulating macromolecules associated with *B. pseudomallei* infection. This technique offers a non-invasive approach to identifying disease biomarkers, opening new avenues for early and accurate diagnosis of melioidosis. It can be exploited to develop a rapid detection method compared to traditional culture methods and can potentially be both specific and sensitive in various clinical and laboratory settings. It holds promise for reducing the mortality rate associated with delayed diagnosis, thereby aiding in reducing the global burden of melioidosis. However, as of now, limited literature and research are exploring the feasibility and accuracy of liquid biopsy. More comprehensive studies and clinical trials are needed to validate the liquid biopsy method’s accuracy, sensitivity, and specificity in this context. Without substantial evidence, the utility of liquid biopsy for diagnosing melioidosis remains uncertain.

#### Circulating cell-free DNA

Circulating cell-free DNA (cfDNA) was first discovered in 1948 in human blood. However, it did not attract much attention until decades later for its applications in infectious disease diagnosis. With technological advancements, many techniques came forward that could harness the potential of cfDNA [55]. Liquid biopsy is one of the techniques that could serve as a powerful tool for the rapid and accurate detection of bacterial DNA, RNA, proteins, or other biomarkers in the body fluids [56], indicating the presence of *B. pseudomallei* infection in the body. cfDNA are smaller than genomic DNA and consist of nucleic acid fragments found in the acellular fraction of the blood and other body fluids. They are thought to originate from the breakdown of dying human cells and microorganisms. When cells undergo apoptosis or necrosis, their DNA is fragmented and released into the bloodstream [57]. Similarly, when microorganisms such as bacteria or viruses die, they also release their DNA into the circulation. This cfDNA can then be detected and analysed, providing valuable information about the presence and nature of infections and other pathological conditions in the body. Although the detection of cfDNA is frequently used in the field of oncology and prenatal diagnostics, clinical applications of cfDNA in infectious diseases are still relatively recent, and not much research has been conducted in this field. Fernández-Carballo BL. *et al*. (2019) reported the potential of cfDNA as a diagnostic tool for detecting pulmonary and extrapulmonary tuberculosis using urine and/or blood samples [55]. Similarly, this approach can be applied to *B. pseudomallei* research. By analysing cfDNA in blood or urine samples, it may be possible to develop a reliable diagnostic method for melioidosis. Such studies could explore the detection of *B. pseudomallei* cfDNA to identify infections, monitor disease progression, and evaluate treatment efficacy.

#### Extracellular vesicles

Extracellular vesicles (EVs) are nanosized lipid bilayer particles released from almost all types of living cells, including bacteria, archaea, fungi, and even eukaryotic cells [58]. These lipid-bound structures can be of variable sizes. The core region of the vesicles consists of a specific cargo reminiscent of their parent cells, and the lipid bilayer has specific protein markers. The cargo of the EVs consists of various bioactive molecules, including selected membrane and cytosolic proteins, DNA, RNA, miRNA (microRNA), and lipids [59,60]. Cargo delivery occurs upon binding specific membrane proteins to their cell receptors; this is followed by fusion with the target cell plasma membrane, after which the luminal cargo is released into the cytoplasm. The specific enhancement of “marker” proteins on EVs facilitates binding to particular targets on specific cells.

Bacterial EVs (BEVs) are released by gram-positive and gram-negative bacteria under all growth phases and environments. Known as the “type zero secretion system,” the difference in structure and cargo of the BEVs is reminiscent of the gram character of the bacteria. They play a crucial role in survival, infection as well as cell-to-cell communication [61]. Some of the prominent biomarkers of bacterial EVs include lipopolysaccharide (LPS) and lipoteichoic acid (LTA) in gram-negative and gram-positive bacteria, respectively [62]. The cargo content of bacterial EV consists of enzymes such as autolysins, periplasmic proteins, nucleic acids, polysaccharides, and peptidoglycan [63]. The proteomic analysis also revealed that the BEVs contain cell wall and carbohydrate metabolism-associated protein and chaperones. Fatty acids such as myristic acid and palmitic acid were identified in BEVs of *B. anthracis* and *S. pneumoniae* [64].

The role of gut microbiota and its effect on metabolic, neurological, and immune systems has recently gained importance. Moreover, the role of microbiota-derived EVs has been a growing concern due to the diverse products that constitute their cargo, thereby affecting the pathways in the host. Depending on their parental bacterial origin, BEVs can be pathogenic or beneficial in patients, leading to various immunological diseases and allergies [65]. For instance, a group of scientists led by Shen et al. demonstrated that *Bacteroides fragilis*-derived EVs harbouring polysaccharide A could be sensed by TLR2 after being transferred to the intestinal dendritic cells, thus mediating anti-inflammatory responses. Additionally, *Bacteroides thetaiotaomicron* EVs carry hydrolytic enzymes that enhance the potential digestion of the gut microbiota by transferring enzymes to bacteria without the hydrolytic enzymes [66]. The cargo content of EVs has microbe-associated molecular patterns (MAMPs), RNA, and DNA that might affect the gut microbiota operating the gut–brain axis by entering the systemic circulation and crossing the BBB (blood–brain barrier) [67]. Although EVs are secreted by nearly all cell types, specific key differences between BEVs and human EVs vividly depict the diversified nature of these vesicles. BEVs carry several pathogenic enzymes and express specific antigens such as polysaccharide A, whereas human EVs carry a more comprehensive range of proteins, a family of miRNAs, etc. In addition to their pathogenic roles, BEVs can be exploited to diagnose infectious diseases and develop therapeutics. BEVs can be utilized as RNAi communication vehicles, drug targeting, and potential vaccine candidates [58]. Hence, BEVs open new avenues for bacterial disease diagnosis and therapy, but nothing much has been explored in the context of *B. pseudomallei*-derived EVs.

### Metabolomic profiling to find unique biomarkers for detection

Metabolomic profiling is a comprehensive analytical approach that can analyse and measure the complete set of metabolites within biological samples [68]. It provides phenotypic information that may not be directly accessible through other omics platforms. This information can be used to make disease diagnoses and prognoses, offering a mechanistic understanding of disease aetiology and the effects of drug treatments [69]. High-throughput spectroscopic and spectrometric technologies are used for biomarker identification in bacterial samples. Lau, S.K.P. *et al*. (2016) reported a non-invasive diagnostic technique for melioidosis using the plasma of melioidosis patients. They carried out metabolic profiling of specific biomarkers using ultra-high-performance liquid chromatography-electrospray ionization-quadrupole time-of-flight mass spectrometry (UHPLC-ESI-Q-TOF-MS) and identified 12 specific metabolites that can be found in significantly elevated amounts in the plasma of melioidosis patients when compared with plasma without any infections as control or plasma from other bacteremia patients [70]. Likewise, in another article, Lau, S.K.P. et al. (2012) reported the use of Matrix-Assisted Laser Desorption Ionization-Time of Flight Mass Spectrometry (MALDI-TOF MS) for rapid identification of *B. pseudomallei* from its related species [71]. In a recent study, Xia L. et al. (2024) investigated the metabolic profiles of melioidosis patients using their plasma samples and kept healthy patients as controls. They identified a 12-metabolite classifier that can distinguish melioidosis from other infectious diseases. Melioidosis non-survivors had significantly elevated levels of some metabolites when compared to melioidosis survivors, including elevated levels of circulating free fatty acids and acylcarnitine and increased valine, isoleucine, and leucine metabolism [72].

## Current status of the market, existing market challenges, and future directions for melioidosis diagnostics

The market for melioidosis diagnostic tests is currently evolving. Although the exact market size data are sparse, it is recognized that the demand for more effective diagnostic tools is increasing, driven by its broader geographical detection and raising awareness of the diseases. The global diagnostic market for melioidosis has also been influenced by technological advancements and the integration of more molecular diagnostics, which promise quicker turn-around time and higher accuracy. Growth factors in this market include increased healthcare expenditure in endemic regions, international collaboration in research and development, and government support for disease control programs to reduce the burden of infectious diseases, including melioidosis. The global market for melioidosis diagnostics is currently segmented into microbiological testing, serological assays, and molecular diagnostics. The market for melioidosis diagnostic kits, while a niche segment and limited, is primarily fuelled by demand in endemic regions. This market is historically constrained by a lack of disease awareness, inadequate healthcare infrastructure, and minimal investment. The segment is characterized by a few companies offering a range of diagnostic solutions, from traditional culture-based methods to rapid diagnostic tests and advanced molecular diagnostics. The largest market share belongs to conventional culture methods, which remain the gold standard despite their limitations in speed and sensitivity due to their high specificity. However, the growing need for rapid diagnostics in emergency and resource-limited settings is driving significant growth in the molecular diagnostics segment.

### Economic analysis of existing diagnostics

The economic landscape of melioidosis diagnostics varies significantly based on the type of diagnostic method employed. The traditional laboratory culture, while being the gold standard, is generally less expensive compared to newer technologies but suffers from longer turnaround times and requires sophisticated lab facilities, which are not always available in endemic regions, specifically in rural areas. The cost for a typical culture test can range from $30 to $100 per sample, depending on regional labour costs and infrastructure [73]. Serological tests and PCR-based methods offer faster results but at a higher cost. PCR tests, for instance, can cost anywhere from $100 to $200 per test, influenced by factors such as royalties for patented technologies, reagent costs, and the type of PCR (quantitative vs. qualitative). The higher sensitivity and specificity of PCR make it a valuable tool despite its higher price point. Several surveys indicate an expected growth of the melioidosis diagnostic test market to reach nearly 25 billion USD in another 3–4 years [74]. Reimbursement scenarios for these diagnostic tests vary by country and insurance policies. In developed countries, insurance typically covers a substantial portion of the cost of advanced diagnostic tests, including PCR. However, in endemic regions like Southeast Asia and Northern Australia, coverage is less comprehensive, which can limit access to the more expensive, though more effective, diagnostic options. Supply chain and logistical issues also play a crucial role in the availability and cost of melioidosis diagnostics. For instance, delays in the supply chain can affect the availability of necessary reagents, particularly in remote areas. Additionally, the requirement for cold chain storage of certain diagnostic components adds a layer of complexity and cost.

### Key players in the market

The market for melioidosis diagnostics includes a mix of biotechnology companies, pharmaceutical firms, and academic institutions focusing on infectious disease research. Some of the leading companies involved in the development and distribution of melioidosis diagnostics include Bio-Rad Laboratories, Thermo Fisher Scientific, and Cepheid, among others. These companies provide diagnostic tools and contribute to research and development in the field. These key players hold significant market share due to their established distribution networks, brand recognition, and comprehensive product portfolios. Strategic alliances, such as partnerships between diagnostic companies and local governments or healthcare providers in endemic areas, enhance their market presence and competitive advantage. These collaborations often aim to improve the accessibility of advanced diagnostics by integrating them into national health programs, ensuring broader market penetration. Table 2 depicts the key players that are manufacture melioidosis diagnostic kits.

**Table 2.** Melioidosis diagnostic kits manufactured by different companies.

Name of the kit	Country of origin	Target gene/ antibody/ antigen	Sample types	Sensitivity	Specificity	Identifier /Developer	Patients recruited	Confidence Interval	NPV: Negative Predictive Value	PPV: Positive Predictive Value	Time (min)	Limit of Detection	Limitations	Ref.
Active Melioidosis-Detect™ (AMD) rapid test	Mahosot Hospital, Vientiane, Lao People’s Democratic Republic	*B. pseudomallei* DNA	Whole blood, plasma and buffy coat, urine, sputum and pus samples	65.4%	87.2%	InBios International, Inc., USA	112	95% CI	89.3% (95% CI 83 to 93.4%)	60.7% (95% CI 45.4 to 74.2%)	15	The concordance rate between AMD and culture was 54.3% (95% CI 36.2 to 72.3%).	It has low overall sensitivity with blood samples and specificity problems with urine samples.	[44]
Hemolysin coregulated protein 1 rapid immunochromatography test (Hcp1-ICT)	Thailand	hemolysin coregulated protein 1	Whole blood samples	74.5%	89.8%	Mahidol University, Thailand	173	95% CI	87.6% (95% CI, 80.3% to 92.5%)	68.3% (95% CI, 55.8% to 78.7%)	10	-	The diagnostic test cannot differentiate between IgG responses from previous and current infections.	[33]
Active Melioidosis DetectTMLateralFlow Assay (AMD-LFA)	India	capsular polysaccharide of *B. pseudomallei*	Urine samples	85.71%	93.62%	InBios International Inc. USA	175	Sensitivity CI: 74.61% to 93.25% Specificity CI: 88.23% to 97.04%	93.62% (CI:88.89% to 96.42%)	85.71% (CI: 75.98% to 91.92%)	–	–	Faint bands in negative urine samples require further evaluation before the test can be adopted as a point-of-care assay.	[29]
IgM and IgG Rapid Cassette Test Kit	Thailand	IgM and IgG antibodies against *B. pseudomallei*	Serum	IgG test: 79% IgM test: 67% IHA test: 72%	IgG test: 90% IgM test: 80% IHA test: 68%	PanBio, Ltd., Windsor, Queens-land, Australia and Wellcome Trust-Mahidol University-Oxford Tropical Medicine Research Programme and Sappasitiprasong Hospital, Thailand	299	95% CI	IgG assay: 91–98%; IgM assay: 85–96%; IHA: 86–96%	IgG assay: 49–79%; IgM assay: 27–59%; IHA: 24–55%.	15	–	The test lacks sufficient sensitivity for standalone diagnosis.	[26]
Real-time PCR test based on type 3 secretion system 1 genes (TTS1-PCR)	Thailand	115-bp orf2 of *B. pseudomallei* TTS1	Buffy coat, plasma, or urine samples.	78.2%	100%	Mahidol University, Thailand	73	95% CI	(95% CI)- 90.7% (84.4–94.6)	(95% CI)- 97.7% (88.2–99.9)	–	–	A limitation of TTS1-PCR is its inability to detect occult cases of melioidosis, which may be identified through the complementary use of Hcp1-ICT.	[33]
Active Melioidosis-Detect™ Rapid Test Kit	USA	capsular polysaccharide (CPS) produced by *B. pseudomallei* and *B. mallei*.	Whole blood, serum, plasma, urine, sputum, pus, bacterial colonies, hemo-culture bottles	80%	100%	In Bios	The kit was primarily tested using bacterial strains	–	–	–		Detection limit comparable to antigen-capture immunoassay; CPS detection in urine > 1.2 × 10^4^ CFU/ml and 50% of serum samples (0.85–6.7 ng/ml).	Extensive preclinical analysis in endemic areas using patient sample is needed to assess the assay’s clinical sensitivity, specificity, and diagnostic utility.	[75]
CRISPR-BP34 Diagnostic Test	Thailand	*B. pseudomallei* DNA	Blood, urine, respiratory secretion, pus, and other body fluids	93%	96.8%	Sunpasitthiprasong Hospital, a tertiary care hospital in Thailand, Mahidol University, Thailand	330	95% CI	–	–	CRISPR-BP34 enables early diagnosis within 4 h to 1 day after patient admission: Blood 1.1 days (IQR 0.7–1.5); Urine 2.3 h (IQR 2.3–2.4); Other fluids 3.3 h (IQR 3.1–3.4)	~50–250 colony-forming units per mL.	It needs scalability and cost reduction for broader use.	[76]
Rapid Antigen Detection Assay	Thailand	*B. pseudomallei* antigen	Pus, pleural fluid, urine and sputum samples	75%	98%	Mahidol University, Thailand	113	–	–	–	Few hours	–	The sensitivity of this test is very low.	[77]
i-STAT Assay	Australia and Cambodia	*B. pseudomallei* capsule	Serum, urine, prostate pus, liver pus, sputum and synovial fluid	Blood: 76% For urine, pus and sputum- High	Blood: 94% For urine, pus and sputum- High	Abbott	299	AUC = 0.91, 95% CI 0.817–1.0)	–	–	10	It can CPS over a > 3 log dynamic range	Its diagnostic potential is limited by the platform used, highlighting the need for more sensitive point-of-care platforms.	[78]
Isothermal recombinase polymerase amplification combined with lateral flow dipstick (LF-RPA) assay	China, Australia, Thailand	Orf2 gene within the putative type III secretion system (T3SS) cluster genes	Blood and soil samples	The sensitivity of the LF-RPA assay was comparable to TaqMan Real-Time PCR	Highly specific	Department of Pestis, National Institute for Communicable Disease Control and Prevention, Chinese Center for Disease Control and Prevention, Changping, Beijing, China	27	–	–	–	30	20 femtogram (fg) (ca. 25.6 copies) of *B*. *pseudomallei* genomic DNA	In this study only mock samples were tested.	[79]
The Active Melioidosis Detect (AMD) lateral flow immunoassay (LFI) and quantitative antigen capture enzyme-linked immunosorbent assay (ELISA)	Lao People’s Democratic Republic (Laos), Asia	Capsular Polysaccharide of *B. pseudomallei*	Serum and urine samples	low	40.5% in urine samples and 6.5% of serum samples	University of Nevada, Reno	34	–	–	–	15	–	Resource-limited endemic areas may lack infrastructure or funding for sample concentration via centrifugation.	[18]
Multiplex serodiagnostic bead assay (BurkPx)	USA	Multiple antigens	Whole blood serum sample	90%	93%	Northern Arizona University and the Menzies School of Health Research	56	95% CI	–	–	–	–	It needs further evaluation in both endemic and non-endemic settings.	[80]
Melioidosis Dipstick (DS) Rapid Test	Thailand	Hcp 1, BPSL2096, BPSL2697 and BPSS0477	serum	92%	97–100%	Mahidol University, Thailand	75	–	–	–	15	–	The multiplex tool’s sensitivity and specificity need re-evaluation in larger, global studies with controls for endemic diseases like leptospirosis.	[81]
Active Melioidosis Detect (AMD) lateral flow immunoassay (LFA)	Australia	*B. pseudomallei* capsular polysaccharide (CPS)	Serum, urine, sputum, pus, plasma and whole blood samples	Serum- 27%, In bacteraemia melioidosis: 39%, In septic shock: 68%; Urine: 63%. In bacteraemia melioidosis: 72%. In septic shock: 90% Sputum: 85% Pus: 83%	High	InBios International, Inc	232	–	–	–	<1 hr	-	Large prospective studies in melioidosis-endemic regions are needed to accurately determine the kit’s specificity.	[82]
16S rRNA Real-Time PCR Assay	Thailand	16S rRNA of *B. pseudomallei*	Blood, throat swabs, sputum/tracheal aspirate, urine, pus or surface swab from wounds and skin lesions	50.9%	99%	Mahidol University, Thailand	383	95% CI	–	–	–	–	The PCR assay lacks sufficient sensitivity to replace culture in clinical settings.	[83]
Melioidosis Real-Time PCR Kit	Thailand	*B. pseudomallei* DNA	Blood	89.6%	96%	Innovation Partner for Impact	–	–	–	–	3 hr	–	A limitation of this kit is its reliance on external collaborations for large-scale production, distribution, and further validation, which may delay widespread accessibility and implementation.	[84]
USM DIAGNOSTIC KIT – Meliodot	Malaysia		blood samples (serum or plasma)	95.5%	92.5%	USM Diagnostic Kit	–	–	–	–	–	–	The kit has not yet been fully commercialized.	[85]
Polyclonal-FITC-based Immunofluorescence Microscopy Assay	Thailand	Monoclonal antibody specific to *B. pseudomallei*	Respiratory secretions, urine, pus and other body fluids	48.4%	99.8%	Mahidol University, Thailand	951	–	–	–	–	2 × 10^3^ CFU/mL.	The assay has high but imperfect specificity, low sensitivity, and still requires culture confirmation for *B. pseudomallei* detection and susceptibility testing.	[86]
TaqMan Real-Time PCR	Malaysia	Orf2 of Type III secretion system (TTSS) gene cluster	Blood and blood culture fluid			University Sains Malaysia	71	–	–	–	–	20 fg (~2.5 copies)	This study is limited by the small number of tested clinical samples.	[87]
*Burkholderia pseudomallei* qPCR Test	Winchester, United Kingdom	*B. pseudomallei DNA*		The kit is designed to have the broadest detection profile possible, and it detects all clinically relevant strains.	Highly specific	YouSeq	–	–	–	–	–	<100 copies of *B. pseudomallei* DNA	This product has been developed for Research use only and is not intended for diagnostic use.	[88]

### Challenges in the current market

#### Technological challenges

Current diagnostic tests for melioidosis, while improving, still face significant technological limitations. Sensitivity and specificity issues are paramount; for example, culture methods, although considered the gold standard, may fail to detect the bacteria in some cases, depending on the stage of the disease and sample quality. Turnaround time is another critical limitation. Traditional culture methods can take several days to yield results, which hampers timely treatment decisions critical in acute infection stages. Compared to culture, the PCR and serological tests offer higher sensitivity but are still susceptible to false positives from closely related bacteria and cross-reactivity issues. Although PCR reduces this time significantly, it still requires hours, and specialized equipment is not readily available in all settings, particularly in low-resource environments.

#### Economic and regulatory challenges

The development and deployment of new diagnostic technologies for melioidosis are fraught with economic and regulatory challenges. The cost of developing new diagnostics, especially those incorporating cutting-edge technologies like next-generation sequencing or point-of-care molecular diagnostics, can be prohibitively high. Furthermore, the market for such tests is relatively small, primarily limited to some geographical regions, discouraging investment from major diagnostic companies. Regulatory hurdles also vary significantly by country and can impede the introduction of new diagnostic tests. For example, any new test must receive FDA approval in the United States, a time-consuming and costly process. In contrast, countries, where melioidosis is endemic, may have less stringent regulatory environments but often lack the infrastructure to conduct necessary clinical trials, creating a paradox where needed diagnostics are harder to approve and deploy.

#### Accessibility and adoption challenges

Distribution and healthcare infrastructure challenges significantly hamper the accessibility and adoption of advanced diagnostics for melioidosis in endemic regions. Many of these regions are rural and have limited access to advanced healthcare facilities. The lack of a cold chain, unreliable power supplies, and insufficient laboratory capacity further restrict the availability of advanced diagnostic methods. Healthcare infrastructure constraints are another major barrier. The scarcity of trained healthcare professionals who can effectively operate sophisticated diagnostic equipment and interpret results accurately exacerbates the problem. Additionally, the initial purchase cost and maintenance of advanced diagnostic equipment remain out of reach for many healthcare facilities in developing countries.

### Future market predictions

#### Advances in artificial intelligence and nanotechnology to aid diagnostic technologies and its impact on market dynamics

The future of melioidosis diagnostic technologies appears promising, with several innovations on the horizon that could transform current practices. Rapid POCTs are anticipated to become more prevalent, facilitating quicker diagnosis directly at the site of patient care. This technology is especially crucial in melioidosis due to the urgent need for timely treatment. Emerging POC tests are likely to employ molecular diagnostics that offer not only speed but also enhanced sensitivity and specificity compared to traditional methods [89]. Further, the integration of artificial intelligence (AI) and machine learning (ML) into diagnostic processes is expected to improve the accuracy of melioidosis diagnostics. AI has effectively addressed intricate problems in several biomedical fields, such as infectious biology, by introducing new ideas and creative solutions [90]. Given the current constraints on early medical diagnosis and the inherent antibiotic resistance of *B. pseudomallei*, AI has the potential to aid in the identification of efficient early detection techniques, the development of new drugs, the targeting of antibiotic resistance, and the creation of a reliable risk prediction and decision support system. AI analyzes extensive datasets to understand the complex patterns of illness transmission in response to climate change, assisting in creating adaptable health interventions [91]. AI algorithms can analyse complex data patterns within X-Ray/CT results, potentially identifying infections earlier and more reliably than current methods. This could lead to more personalized treatment plans and better patient outcomes. While the implementation of AI in addressing melioidosis difficulties is currently limited, Machine Learning (ML), a fundamental branch of AI, and other AI techniques are increasingly employed for diagnosing many infectious illnesses and have the potential to be applied to melioidosis. AI-driven techniques facilitate ligand-based drug design and the identification of novel therapies by forecasting and producing dynamic molecular surface interaction fingerprints, enabling informed drug design opportunities for targeting melioidosis. Over time, several advanced bioinformatics algorithms have been developed to predict antimicrobial resistance genes (ARGs). The Comprehensive Antibiotic Resistance Database (CARD) is a platform designed to precisely identify Antibiotic Resistance Genes (ARGs). The recently published bioinformatics methodology, validated by ISO [92], aids laboratory personnel in identifying antimicrobial resistance (AMR). Nevertheless, AI plays a crucial role in advancing research on AMR and has shown potential in controlling AMR, which can also be extended to melioidosis. AI may be used to examine patient data, including demographic details and medical records, forecast the likelihood of melioidosis, and assist in choosing suitable preventive or curative measures.

Nanotechnology has made significant progress in developing biosensors, which are devices used to detect biological substances. Researchers have also been exploring new nanomaterials to create these biosensors. Nanotechnology-based biosensors hold the ability to convert biological interactions into detectable and quantifiable electric signals, which is their greatest strength. Accurately studying minute differences in biological processes when mixed biomolecules is also vital. These advancements can potentially be used in the field of melioidosis [93]. Upcoming technologies are poised to disrupt the current market dynamics significantly. As new players with innovative diagnostic solutions enter the market, traditional companies might be challenged to maintain their market share unless they adapt quickly to the evolving landscape. For instance, startups focusing on AI-driven diagnostics or portable POCT devices could quickly ascend to leadership positions by offering cost-effective, accurate, and user-friendly alternatives to conventional methods. These technological advancements will likely alter the competitive environment, making it more dynamic and diversified. Traditional market leaders must invest heavily in research and development or form strategic partnerships with tech startups to stay relevant. Additionally, the increasing role of digital health could see technology companies becoming critical players in the diagnostics market, further shifting the traditional dynamics and introducing new potential leaders focused on innovative approaches.

#### Biosensor technology

Biosensors are analytical devices that combine a biological sensing element with a transducer to detect and quantify biological or chemical substances. They offer numerous advantages, including high sensitivity, specificity, rapid response times, and the potential for miniaturization and portability. Biosensors are employed in various fields, such as medical diagnostics, environmental monitoring, food safety, and biotechnology. The integration of advanced materials, nanotechnology, and microfluidics has significantly enhanced the performance and applicability of biosensors, making them invaluable tools for real-time and point-of-care (POC) diagnostics [94].

##### Metal oxide-based biosensors

Metal oxide-based biosensors have attracted considerable attention due to their unique properties, such as high electron mobility, chemical stability, and biocompatibility. These biosensors utilize metal oxides like zinc oxide (ZnO), titanium dioxide (TiO₂), and tin oxide (SnO₂) to enhance sensitivity and specificity in detecting various pathogens, including bacteria, viruses, and other biomarkers. Metal oxides can be synthesized into various nanostructures, such as nanowires, nanorods, and nanoparticles, which provide a large surface area for the immobilization of bioreceptors [95].

##### Zinc nanoparticle-based biosensors

The ZnO nanoparticle-based biosensors are particularly promising for detecting bacterial diseases, including melioidosis. ZnO nanoparticles offer several advantages, including high surface-to-volume ratio, excellent biocompatibility, and strong adsorption capability for biomolecules. These properties make ZnO nanoparticles ideal for enhancing the performance of biosensors. ZnO nanoparticles can be functionalized with antibodies, aptamers, or DNA probes to specifically detect *B. pseudomallei*, the causative agent of melioidosis. The use of ZnO nanoparticles in biosensors has been shown to significantly improve sensitivity and detection limits, enabling the identification of bacterial DNA or antigens at very low concentrations. For example, ZnO nanoparticle-enhanced electrochemical biosensors have demonstrated the ability to detect *B. pseudomallei* DNA with high specificity and sensitivity [96].

##### Sample type and biosensor technology

In recent years, biosensor technology has emerged as a promising solution to address these challenges by providing rapid, sensitive, and specific detection of *B. pseudomallei* [11]. For the effective detection of melioidosis using biosensors, selecting appropriate clinical samples is crucial. Suitable samples for targeting include blood, serum, urine, and respiratory secretions. Blood and serum samples are particularly useful for detecting bacteraemia and septicaemia, common manifestations of melioidosis. However, targeting non-invasive samples such as sputum and urine is particularly advantageous for patient comfort and compliance.

Sputum samples are valuable for diagnosing respiratory manifestations of melioidosis, such as pneumonia. The collection of sputum is non-invasive and can be done easily, making it suitable for frequent monitoring. Biosensors designed to detect *B. pseudomallei* in sputum can provide rapid and accurate results, facilitating timely diagnosis and treatment. For example, the use of electrochemical biosensors that detect bacterial DNA or specific antigens in sputum has shown promise in enhancing diagnostic capabilities [97]. Urine samples offer a non-invasive option for detecting melioidosis, particularly in cases where the bacteria have disseminated through the bloodstream. The presence of *B. pseudomallei* or its components in urine can be detected using biosensors with high sensitivity. The integration of nanomaterials, such as gold nanoparticles, in biosensors has improved the detection limits, enabling the identification of bacterial DNA or antigens at very low concentrations. This approach is advantageous for monitoring disease progression and assessing the efficacy of treatment [44]. Non-invasive samples like sputum and urine are particularly appealing for continuous monitoring and early detection, making them ideal candidates for biosensor applications in melioidosis diagnostics.

##### Recent advancements in biosensor technology for Melioidosis

Recent advancements in biosensor technology have focused on enhancing sensitivity, specificity, and portability to facilitate POC diagnostics for melioidosis. One significant development is the integration of flexible microfluidic platforms with biosensors. This approach simplifies the fabrication process and reduces costs, making it feasible for one-time use to prevent disease transmission. The use of paper-based flexible microfluidic biosensors that leverage the capillary action for fluid manipulation without external pumps or valves is a key POCT platform. This innovation not only lowers production costs but also enhances the usability of biosensors in resource-limited settings [98]. Another noteworthy advancement is the development of electrochemical biosensors, which offer high sensitivity and rapid response times. These sensors utilize various transduction methods, such as amperometric, potentiometric, and impedimetric techniques, to detect the presence of *B. pseudomallei* antigens or DNA. Recent studies have demonstrated the effectiveness of these biosensors in detecting low concentrations of bacterial targets, making them suitable for early-stage diagnosis [99].

Additionally, the application of nanotechnology has significantly improved biosensor performance. Nanomaterials, such as gold nanoparticles, carbon nanotubes, and quantum dots, have been employed to enhance signal transduction and increase the surface area for bioreceptor immobilization. These nanomaterial-based biosensors exhibit superior sensitivity and specificity, enabling the detection of *B. pseudomallei* at extremely low levels. For instance, a study demonstrated the use of gold nanoparticle-enhanced biosensors for detecting *B. pseudomallei* DNA, achieving a detection limit of 10 femtomolar [99].

##### Current challenges in biosensor technology

Despite these advancements, several challenges remain in the development and deployment of biosensors for melioidosis. One of the primary challenges is ensuring the specificity of biosensors in complex biological matrices. The presence of interfering substances in blood, serum, or other clinical samples can lead to false positives or negatives, compromising the accuracy of the diagnosis. Researchers are working on improving the selectivity of biosensors through the use of highly specific bioreceptors, such as monoclonal antibodies or aptamers, and implementing advanced signal processing algorithms to filter out noise. Another challenge is the stability and reproducibility of biosensors. Environmental factors, such as temperature and humidity, can affect the performance of biosensors, particularly those that rely on biological recognition elements. Ensuring the long-term stability of these sensors is critical for their practical application in field settings. Efforts are being made to develop more robust biosensors using synthetic bioreceptors and protective coatings to enhance their durability under varying environmental conditions [100]. The integration of biosensors with user-friendly POC devices also poses a challenge. While significant progress has been made in miniaturizing biosensors, developing complete POC systems that include sample preparation, signal processing, and data interpretation remains a complex task. Collaborative efforts between researchers, engineers, and clinicians are essential to design and validate integrated POC platforms that can deliver reliable and actionable results in real-time [101].

#### Market growth and expansion predictions

The market for melioidosis diagnostics is projected to grow significantly over the next decade. Several analysts predict an annual growth rate of approximately 5–7%, driven by increased awareness of the disease, improvements in healthcare infrastructure, and the introduction of advanced diagnostic technologies. This growth is also supported by expanding into new geographical markets, especially in areas recently identified as emerging disease hotspots, including parts of Africa and South America.

#### Opportunities for new entrants and investors

##### Investment opportunities

The melioidosis diagnostics market offers diverse investment opportunities that can yield significant returns while contributing to public health improvements, especially in underserved areas. Investing in developing rapid, accurate, and cost-effective diagnostic technologies, such as POCTs and molecular diagnostics, can meet a critical market need. There is also considerable potential for exploring AI and ML applications to enhance diagnostic accuracy and predictive capabilities. Expanding diagnostic solutions to new geographic markets, particularly in regions recently identified with rising cases of melioidosis, like parts of Africa and South America, can fill significant healthcare gaps. These markets are relatively untapped and could benefit significantly from enhanced diagnostic capabilities. Investments to enhance laboratory capacities, train local healthcare workers, and establish better supply chain logistics in endemic regions would aid in better disease management and support broader market penetration strategies.

##### Strategic recommendations for new market entrants

Focus on developing solutions for unmet needs within the market, such as ultra-rapid diagnostics, more reliable serological tests, or cost-effective mass screening tools. Niches can also be found in creating tailored solutions for specific regional challenges, such as tests that perform well in varying environmental conditions or that can be integrated into existing healthcare practices with minimal disruption. Strategic partnerships with existing players, like major diagnostic firms, or collaborations with academic and research institutions can provide new entrants with essential market insights, technology transfer opportunities, and regulatory guidance. These relationships can also facilitate pilot testing and adoption in target markets, easing market entry. Understanding and navigating the regulatory landscape is crucial. New entrants should invest in regulatory expertise early in their development process to ensure local and international standards compliance. This approach can expedite market entry and establish credibility. Utilizing regulatory consultants and partnering with local entities can also streamline this process.

## Challenges in melioidosis diagnosis

Given the disease’s high mortality rates, especially when treatment is delayed, the role of timely and precise diagnostics cannot be overstated. Current diagnostic methods are critical for clinical decision-making and informing public health strategies to control and prevent the disease, particularly in endemic regions. One of the pertinent challenges is technical limitations regarding sensitivity and specificity. The reliability of diagnostic tests for melioidosis varies significantly, with sensitivity and specificity fluctuating across different methods such as culture, serology, and PCR methods, considered the gold standard, have high specificity but variable sensitivity, which can lead to underdiagnosis or misdiagnosis. For instance, culture, the gold standard, is time-consuming and requires a biosafety level 3 laboratory. While useful for screening, serological tests often suffer from cross-reactivity with other bacteria and Burkholderia species, reducing their specificity. Though PCR offers improved sensitivity and specificity, it is not universally available in all settings, predominantly rural areas. The variability in these test characteristics can significantly impact clinical outcomes and disease management. Misdiagnosis or delayed diagnosis due to low sensitivity can lead to inappropriate treatment, while false positives due to low specificity can cause unnecessary anxiety for the patient and wrong treatment.

Another critical challenge in melioidosis diagnosis is the turnaround time and technological barriers. Rapid diagnosis is crucial for effective treatment of melioidosis, which can progress rapidly, especially in cases of septicaemia. Traditional culture methods, while accurate, can take several days to yield results, which is suboptimal in acute and emergency scenarios. Modern rapid tests, such as PCR-based assays, offer quicker results but are often unavailable in many endemic regions due to cost and infrastructural demands. Additionally, the operation of such equipment typically demands a high level of technical expertise and training that local healthcare personnel might not possess. Setting up, maintaining, and buying PCR equipment and consumables may be expensive and a barrier for many healthcare facilities, particularly those in lower-income countries where melioidosis is most prevalent. The financial challenges associated with advanced melioidosis diagnostics are compounded by issues related to insurance and reimbursement, particularly in underdeveloped countries where insurance coverage is not guaranteed. The direct costs to patients for advanced diagnostic tests can be considerable when not covered by insurance. This gap necessitates either significant training programs or reliance on more straightforward, albeit less effective, diagnostic methods.

Another bottleneck is the complex regulatory landscape and approval barriers for new diagnostic tests, which can vary significantly from one country to another, influenced by varying national healthcare policies, standards, and priorities. New diagnostic tests must undergo regulatory reviews of their safety, effectiveness, and quality before being licenced for clinical use. This strategy includes preclinical investigations, clinical trials, and comprehensive documentation. The regulatory structure or resources needed to accelerate diagnostic approval may be lacking in melioidosis-endemic low-income countries. Moreover, meeting international diagnostic test development and implementation standards is another significant barrier, especially for newer and more innovative technologies. The requirements for detailed documentation, extensive testing, and quality assurance protocols can be resource-intensive and beyond the capabilities of smaller or less-funded developers. This compliance is crucial for gaining access to international markets, especially in the European Union or the United States, where regulatory requirements are rigorous. Non-compliance can restrict the geographical reach of innovative diagnostics, limiting their impact on global health.

## Future directions

Early and precise diagnosis directly impacts clinical outcomes, reducing the high mortality rates associated with the disease, especially in endemic regions. Furthermore, with the increasing global spread of *B. pseudomallei* due to environmental changes and human migration, developing more effective diagnostic tests becomes imperative for global health security. As such, future advancements in diagnostic technologies must aim to enhance the sensitivity and specificity of the existing methods, reduce dependency on high-level biosafety facilities, and promote the development of rapid POCTs that can be used in resource-limited settings. These improvements will facilitate better disease management and enable more robust surveillance and control measures, helping curb the spread of this potentially deadly infection. Recent technological advancements in developing POCS and molecular diagnostics are crucial in managing melioidosis [80]. Advancements in PCR and Next-Generation Sequencing (NGS) represent a transformative sted. NGS allows for the comprehensive characterization of the pathogen at the genetic level, facilitating accurate diagnosis and insights into antibiotic resistance patterns, virulence factors, and epidemiological typing. Another aspect is ensuring test results’ reliability, global standardization, and rigorous validation protocols. International collaborations can facilitate the standardization of methodologies and criteria for validation, ensuring that diagnostic kits are effective across different regions and populations.

**Figure 7.**
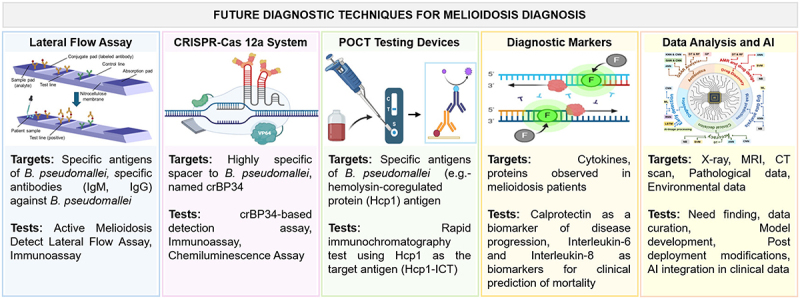
Future of melioidosis diagnostic techniques. Future diagnostic advancements include point-of-care diagnostics, including lateral flow assays, which provide rapid results and are particularly useful in resource-limited settings. CRISPR-Cas-based assays, diagnostic biomarkers, and AI models are also expected to enhance detection speed, accuracy, and accessibility, addressing current limitations and improving early diagnosis in endemic regions.

Integrating advanced computational techniques like AI, ML, and IoT in melioidosis diagnostics holds promise for revolutionizing this field. AI and ML can help analyse complicated information faster than conventional approaches to improve diagnosis accuracy. ML models may predict clinical outcomes based on data patterns, aiding early diagnosis and personalized therapy. AI algorithms are also being developed to help physicians analyse NGS data, making these complex analyses more accessible and useful. Advancements in multi-omic technologies, AI, and data analysis tools can refine diagnostic tests by improving assay designs to distinguish between *B. pseudomallei* and closely related non-pathogenic species. Another important aspect in advancing melioidosis’s diagnostic capabilities is identifying and developing novel biomarkers [82]. Research focused on discovering unique genetic, proteomic, or metabolic markers specific to *B. pseudomallei* can lead to the development of tests that are not only faster but also more accurate. These biomarkers could potentially be used to detect the presence of infection even before the onset of symptoms, which is crucial in preventing the progression of the disease to its more severe forms. Once identified, these novel biomarkers must undergo rigorous validation and standardization to ensure their effectiveness and reliability in a clinical setting. Standardization involves the creation of protocols for the biomarker’s detection and quantification, ensuring that tests developed can be universally applied and yield consistent results across different laboratories and conditions. Developing novel nanomaterials and nano-based biosensor technologies can help enhance the sensitivity and specificity of existing diagnostic methods, which is crucial for improving the detection and management of melioidosis. The advent of micro and nano-level electronics can help in cost-reduction strategies, improving the economic accessibility of diagnostic tests for melioidosis in both developed and endemic regions. Innovations in test design, such as developing multiplex assays or using less expensive materials, can substantially decrease the cost per test. The future melioidosis diagnostic techniques are summarized in Figure 7.

Additionally, localized production of diagnostic kits in endemic regions can reduce logistical expenses and make advanced diagnostics more affordable. This approach supports local economies and ensures a more sustainable supply of diagnostic tools in regions most affected by the disease. Furthermore, advocacy for policy changes that support broader financial support for diagnostic testing can lead to more comprehensive healthcare systems, allowing for earlier detection and treatment of melioidosis, essential for improving patient outcomes and reducing overall healthcare costs. Another critical aspect is integrating global regulatory networks, streamlining approval processes, and compliance with international guidelines, which suggests harmonizing international cooperation. There is a crucial need for enhanced training plans and diagnostic infrastructure to manage and diagnose melioidosis, especially in endemic regions [89]. Continuing education initiatives are essential to ensure healthcare professionals stay updated with ongoing technological advancements and procedural updates in melioidosis diagnostics. Such educational efforts can improve diagnostic accuracy and efficiency, improving patient management and outcomes. Moreover, establishing industry-academia partnerships and Global Health Initiatives can strengthen the fight against melioidosis. Joint ventures drive technological innovation and facilitate the translation of research findings into practical, market-ready solutions that can be rapidly deployed in affected regions. Engagement with international health organizations is crucial for amplifying the impact of diagnostic improvements on a global scale. Organizations, such as the World Health Organization (WHO), Centres for Disease Control and Prevention (CDC), and various Non-Governmental Organizations (NGOs), can provide platforms for the dissemination of new diagnostic methods and best practices. Collaborative projects under these umbrellas often focus on disease surveillance, data sharing, and management improvements, enhancing the overall response to melioidosis outbreaks.

## Conclusion

Melioidosis, the disease caused by *B. pseudomallei*, significantly affects the human population. The disease has been neglected even after having a significantly higher rate of mortality and morbidity. Patients having other comorbidities like diabetes, compromised immune system, alcoholism, renal disease, etc., are more prone to fatality post-infection. Misdiagnosis, underdiagnosis, and late diagnosis play a major role in complicating the disease severity. Presently, the culturing of bacteria is said to be the gold standard for diagnosis, but this method is time-consuming as the growth of bacteria on the culture is slow. *B. pseudomallei* is misdiagnosed or not correctly diagnosed for several reasons, such as sometimes it is considered a laboratory contaminant, leading to delayed or wrong treatment. The major reasons behind misdiagnosis are lack of awareness about the severity of the disease, unavailability of proper diagnostic tools, and so on, specifically in remote areas. The current diagnostic techniques for melioidosis face several challenges in terms of accuracy and specificity. As the understanding of the pathogenesis of melioidosis continues to evolve, it is crucial to develop POCT molecular diagnostics and infrastructure to enhance the specificity and sensitivity, which is economical and easily accessible even in remote areas for an efficient diagnosis and treatment. In addition, collaborations between researchers, clinicians, and industry partners using advanced technologies like AI, ML, IoT, nanotechnology, sensor technology and their integration is pivotal in driving the translation of novel diagnostic technologies from the laboratory to clinical practice. Finally, programs like global health initiatives, workforce training, cost reduction strategies, and strengthening surveillance and public health infrastructure will combat disease’s adverse effects on humans.

## Data Availability

Data sharing is not applicable to this article as no new data were created or analysed in this study.
